# Axonal Injury with Persistent Neuropathy following Popliteal Nerve Block for Cheilectomy Surgery

**DOI:** 10.1155/2021/9942195

**Published:** 2021-07-05

**Authors:** Y. A. M. Kuijpers, J. M. Setz, K. Khemlani-Houthoff

**Affiliations:** ^1^Department of Anesthesia, Maxima Medical Centre, Veldhoven, Netherlands; ^2^Department of Neurology, Maxima Medical Centre, Veldhoven, Netherlands

## Abstract

Peripheral nerve blocks are often used for foot and ankle surgery. The occurrence of persistent neurological symptoms thereafter is very rare. Preventive strategies pose no guarantee and uncovering true etiology is often complicated. We discuss a case in which a young, healthy patient developed nerve damage after an uneventful popliteal block and cheilectomy. Nerve conduction studies revealed axonal injury in the distribution area of the sciatic nerve. The neurological symptoms persisted for more than 12 months, emotionally affecting the patient greatly. Patients will primarily report to the orthopedic surgeon, for whom cooperation with anaesthesia and neurology is of importance. Anesthetic involvement probably improves patient satisfaction during complication management.

## 1. Introduction

Foot and ankle surgery is often accompanied by peripheral nerve blocks (PNBs) for perioperative pain management. Long-term peripheral nerve injury (PNI) is an uncommon complication, but could be reported at postoperative check-up at the orthopedic outpatient clinic. Depending on definitions, the incidence for neurologic symptoms >6 months ranges from 0 to 0.7%, specifically for popliteal nerve blocks [1, 2]. Available literature states that perioperative nerve damage has a multifactorial etiology, with only one-third of the PNI actually associated with PNBs. For educational purposes, we describe a case in which postoperative neurological symptoms developed due to axonal injury. Additional literature on etiology, preoperative preventive strategies, and postoperative diagnostics is discussed. Most importantly, we emphasize the importance of a multidisciplinary approach, timely consulting the anaesthesiologist and neurologist in the postoperative course. The patient had provided written consent for the publication of the case.

## 2. Case Description

A woman in her late thirties, presented to the orthopedic outpatient clinic. Her history revealed a right-sided ankle trauma 5 years ago and a pulmonary embolism/deep venous thrombosis (DVT) while on oral contraceptives 17 years ago. Current consultation was for right-sided hallux rigidus, with metatarsophalangeal joint osteoarthritis. A cheilectomy was planned. She was considered ASA II status and currently did not use any medication. The anesthetic plan consisted of general anaesthesia combined with a popliteal nerve block for postoperative pain relief. Informed consent was obtained. Preoperatively, the patient underwent ultrasound guided, with use of a neurostimulator (0.5 mA, 0.3 ms, 2 Hz), single-shot popliteal block. No sedatives were administered. A 22 G Braun Stimuplex® Ultra 360® with a 30-degree angled needle tip was used. The procedure was executed by an anaesthesiologist in training under direct supervision of an experienced consultant anaesthesiologist. A total of 20 cc ropivacaine 0.75% without additives was injected perineural with subjectively low injection pressure. Injection was nonpainful. The procedure was unremarkable and the nerve block spread accordingly. General anaesthesia was induced with sufentanil and propofol. After induction, dexamethasone (8 mg) and granisetron (1 mg) were administered. Placement of a supraglottic airway device was uneventful and anaesthesia was maintained with sevoflurane. During surgery, the patient was in supine position with a tourniquet around the right upper leg at a pressure of 250 mmHg for 18 minutes. Mean arterial pressure always exceeded 70 mmHg, without the use of vasopressor agents. The cheilectomy procedure went surgically uneventful.

Eight days after surgery, the patient had persistent numbness and paresthesia of the foot, initially attributed to postoperative hematoma and swelling. After two weeks of progressive complaints, the neurologist was consulted. Physical examination revealed hypesthesia in the toes, ball, and lateral part of the foot and lateral part of the lower leg. Theoretically, the area is related to the superficial peroneal, tibial, and sural nerve. Loss of motor function was not objectified, although subjective weakness of toe-extension existed. Additionally, she mentioned an invalidating painful cramping of the calf musculature during physical activity. Postoperative PNI after PNB was suspected and nerve conduction studies and electromyography (EMG) were conducted. This revealed absent sensory conduction of the superficial peroneal nerve on the right side. Conduction velocity of the right sural nerve was normal. Compound muscle action potential amplitudes of the right extensor digitorum brevis muscle were decreased, with also denervation potentials seen during myography. The amplitudes of the right abductor hallucis brevis muscle were also decreased. Therefore, the conclusion of the EMG was a partial sciatic nerve neuropathy with axonal injury. Three months after surgery, the patient still experienced complaints and was emotionally affected by the series of events, filing an informal complaint towards the anaesthesia department. It was at this point that the anaesthesiologist, involved in the case, was informed. During follow-up at 5 and 10 months after surgery, the EMG showed recovery and also clinical recovery was present. Despite improvements 12 months after surgery, the patient received continued counseling from a physical therapist, still reporting daily complaints associated to the nerve damage.

## 3. Discussion

Long-term peripheral nerve injury (PNI) is an uncommon complication, with an aforementioned incidence of 0–0.7% for popliteal nerve blocks. The benefits of nerve blocks for perioperative pain management in bone and joint surgery are therefore greater than the risks. A divergent postoperative course would present during postoperative check-ups at the orthopedic outpatient clinic, outside of the anaesthesiologist's view. However, the impact on patients, if PNI does occur, might be enormous as it was for our patient. Patients might impute their symptoms to the PNB, although literature implies multifactorial etiology. Anesthesiologists should be able to provide explanation, based on current knowledge, and guide the patient postoperatively. If association of neurologic symptoms with a conducted PNB is likely, several pathogenic pathways such as mechanical, chemical, pressure, or vascular damage might contribute [3, 4]. Chemical damage is proven for all local anesthetics (LA), as they show cytotoxicity in vitro and in vivo, inducing histological damage and metabolic alterations, resulting in cell death and apoptosis. This is thought to be a consequence of prolonged intracellular Ca^2+^ concentrations [3]. The clinical symptoms of neurotoxicity can vary from neurological deficits to neuropathic pain and may be observed for months after initial injury. Concentration, duration of exposure, and location of deposit influence the extent of toxic damage [3]. Besides neurotoxicity, vascular compromise can induce nerve ischemia. Especially after intraneural injection, sustained high intraneural pressures can exceed capillary occlusion pressures of the vasa nervorum [4]. However, when LA was applied topically on the exposed sciatic nerve in rats, all showed vasoconstrictive properties, with ropivacaine showing the greatest decrease in neuronal blood flow [5].

Anesthesiologists use multiple measures to try to prevent nerve injury. The purpose of these measures is avoidance of intraneural (and especially intrafascicular) injection, leading to axonal injury. Objectifying needle-tip placement is herein the subject of interest. Neurostimulation is often used to assess needle-to-nerve contact although correlated sensitivity is low [6]. Additional ultrasound guidance has advantages for needle localization and reduces the risk of systemic LA toxicity during PNB procedures. However, based on historical cohorts, it does not reduce the risk of persistent long-term PNI compared to neurostimulator-based nerve localization alone [7]. Moreover, ultrasound might identify intraneural needle placement, but cannot distinguish between extra- and intrafascicular needle placement. Clinically, intrafascicular injection can induce high injection pressures. Subjective pressure estimation is proven to be unreliable, but there are several, simple and more advanced, methods to objectify injection pressures. Evidence suggests that opening pressures <15 PSI correlate with extrafascicular needle position. However, no evidence to date has proven rigorous reduction of PNI [8]. When accidental intraneural needle placement occurs, a high gauge, short beveled needle significantly reduces the risk of perforation of fascicles. In our case, a 22 G and 30-degree angled needle tip was considered safe [9]. The occurrence of nerve damage is not predictable. Even intentional intraneural injection did not result in higher incidences of PNI [10]. Clinically uneventful procedures simply do not imply uneventful recovery, and vice versa. With the current knowledge and materials, it might be a difficult undertaking, if not impossible, to decrease the incidence of PNI even more by only operator chariness. Unfortunately, since persistent neurological injury after PNB is rare, generating sufficient scientific evidence to resolve this question will be challenging. When postoperative PNI is suspected, nerve conduction studies are the cornerstone of diagnosing nerve damage [11]. It can be helpful, but it cannot prove etiology. For example, when routinely performing EMG after intentional intraneural injection during PNB, electrophysiologic abnormalities indicating some degree of axonal damage were found in 100% of nonsymptomatic patients [10]. Moreover, EMG cannot differ between a singular lesion proximal and two distinct lesions more distally, when not anticipated for. For our patient, symptoms in the distribution area of the superficial peroneal nerve were clinically most evident and were the main finding on EMG. This may be independent of the PNB. The aforementioned painful cramps in the calf were not explained by EMG. Furthermore, EMG cannot always differ in timing of lesions, which could be of importance since prior ipsilateral ankle trauma and DVT might have influenced distal nerve integrity. Factors such as the PNB, use of a tourniquet, and direct surgical damage might have contributed as a “second hit.” It should be noted that nerve injury is also the most common complication associated with the use of a tourniquet. The pathophysiology remains unclear, but it is likely that mechanical compression and neural ischemia are both involved [4]. As mentioned above, neural ischemia may also be caused by intraneural LA deposition and vasoconstrictive properties of LA. A combination of these mechanisms might conceivably influence the adequate reperfusion after tourniquet deflation.

When performing EMG early (7 days to 3 weeks) after suspected injury, signs of acute denervation may be visible suggesting recent damage [10]. EMG performed later will mostly show reinnervation signs. In the current case, the anaesthesia department was consulted quite late in the course of the case. Benefit of earlier consultation might be found in advice for testing specific locations of possible nerve damage, timing, and the precise clinical question. The anaesthesiologist should therefore be familiar with the possibilities and value of nerve conduction studies [11]. Nerve injury had a major impact on the patient in the current case and emotions such as sadness and anger occurred. Explanation and patient support by anaesthesia might help patients feel heard and taken seriously and provide understanding, even though actual causality may not be present. Unfortunately, anesthesiologists probably often miss out on (temporary) neurological symptoms after surgery.

## 4. Conclusion

Although long-term PNI is an uncommon complication, it can have devastating consequences for the patient. Anesthesiologists take multiple measures to avoid PNI. Postoperative communication between the anaesthesiologist, orthopedic surgeon, and neurologist is important to start the appropriate diagnostic pathway when PNI is suspected. This may also contribute to the patient feeling heard and taken seriously, based on open communication. Postoperative check-up is done by orthopedic colleagues and we would like to address the importance of timely consultation of the anaesthesiologist, especially for patient satisfaction.
